# Supplementary material for: Charge-specific size-dependent separation of water-soluble organic molecules by fluorinated nanoporous networks
Source: Nat Commun. 2016 Nov 10;7:13377. doi: 10.1038/ncomms13377 (PMC5109553; doi:10.1038/ncomms13377)
Supplement: Supplementary Information — Supplementary Figures 1-17, Supplementary Table 1, Supplementary Notes 1-3 and Supplementary References. [file ncomms13377-s1.pdf]

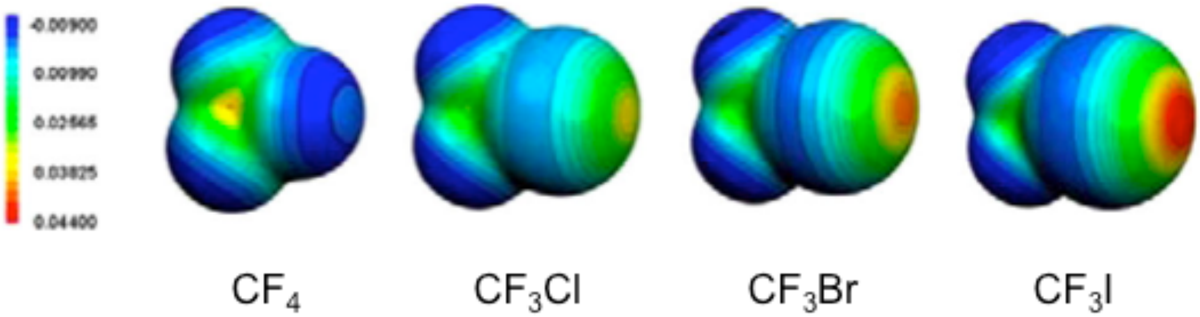

Supplementary figure 1 | Molecular electrostatic potential surface of CF_3X. Reprinted with permission from Supplementary Reference #4. Copyright 2007 Springer.

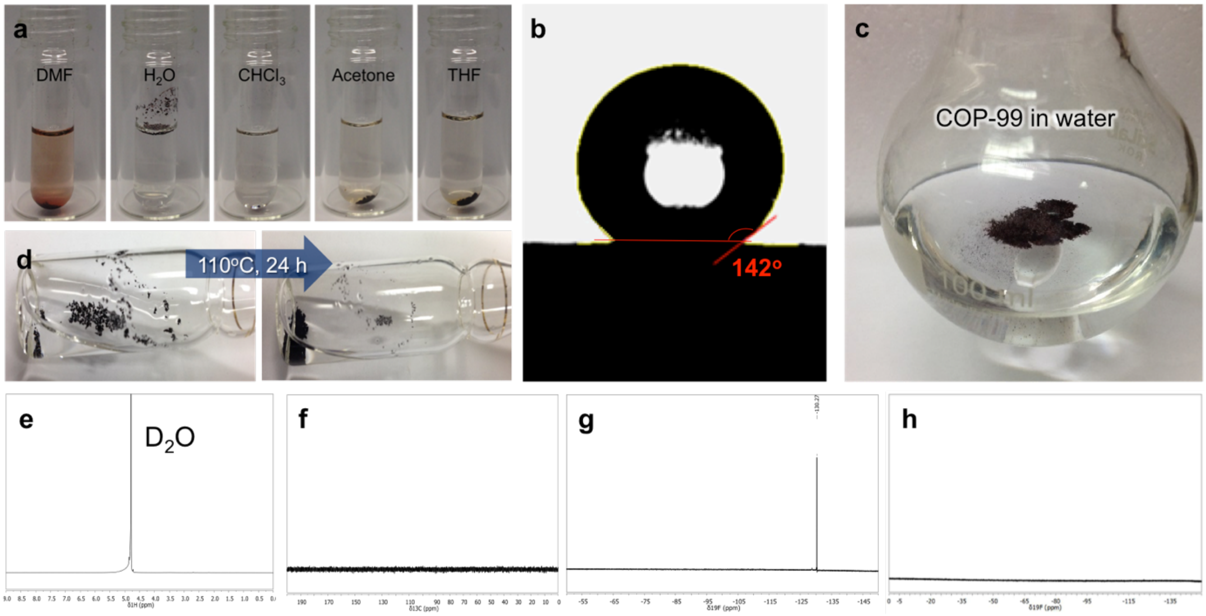

Supplementary figure 2 | Stability tests for COP-99. (**a**) Solubility test of COP-99 in common solvents. Images were taken after a sonication for 1 min at 50 ºC. (**b**) Contact angle for a water droplet on the surface of COP-99. (**c**) Photograph of COP-99 in water. Owing to the hydrophobicity, COP-99 floats on the surface of water. (**d**) Long-term boiling test of COP-99 (20 mg) in D_2O (1.5 mL) at 110ºC for 24 h. In order to prevent D_2O evaporation, the test was conducted in a sealed glass ampoule. There was no noticeable change in both COP-99 and D_2O solution after boiling. (**e**) ^{1}H and (**f**) ^{13}C NMR spectrum of the D_2O filtrate. (**g**) ^{19}F NMR spectrum of D_2O the filtrate exhibited the existence of free fluorine partially detached from COP-99 via nucleophilic substitution. (**h**) Fluorine peak was not observed when the COP-99 was treated in D_2O at 50 ºC for 60 h.

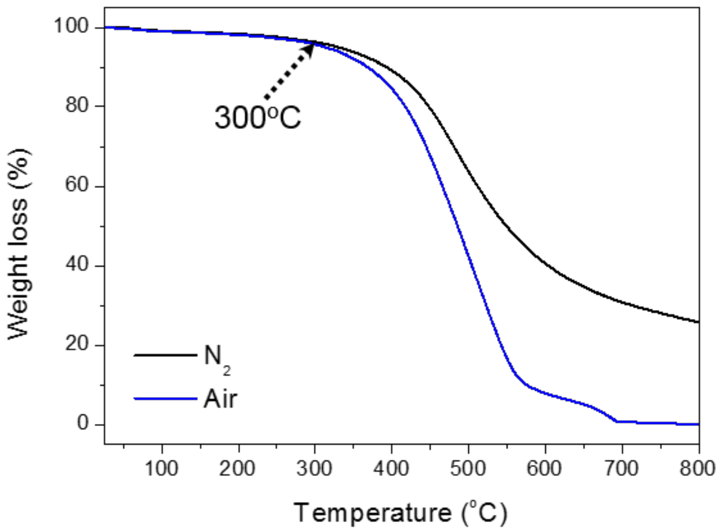

Supplementary figure 3 | Thermogravimetric analysis (TGA) plot of COP-99. COP-99 shows high thermal stability up to 300ºC both in inert and oxidative environments.

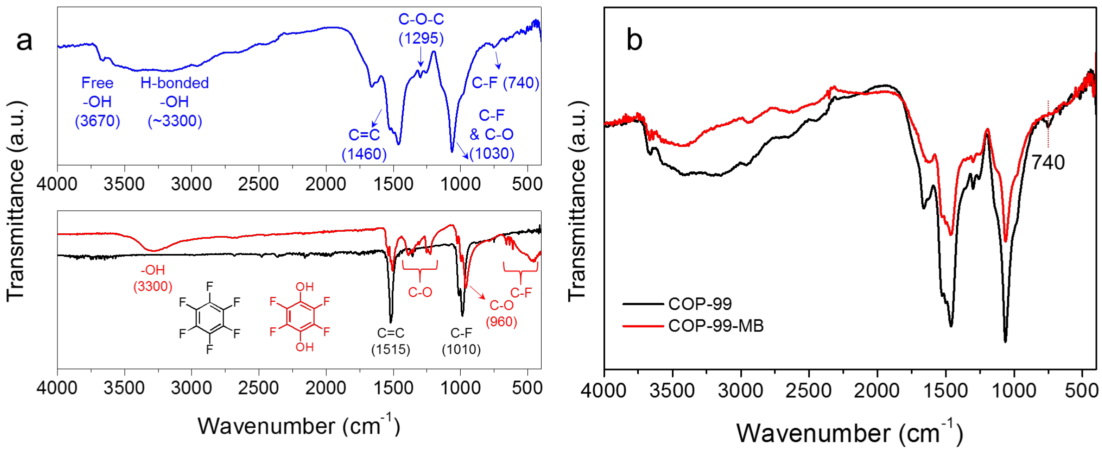

Supplementary figure 4 | Characterisation by FTIR. (**a**) FTIR spectra of COP-99 and related monomer units. (**b**) Change in FTIR spectrum after the adsorption of MB molecule.

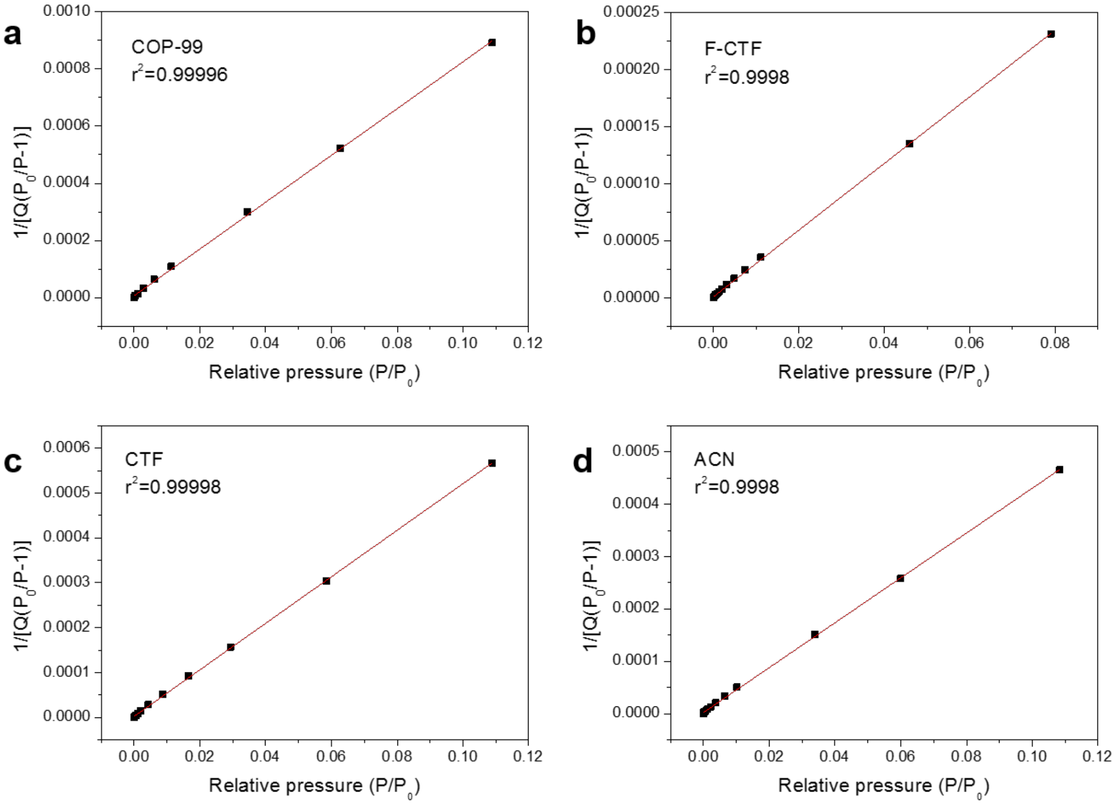

Supplementary figure 5 | BET linear plots of (**a**) COP-99, (**b**) F-CTF, (**c**) CTF, and (**d**) ACN.

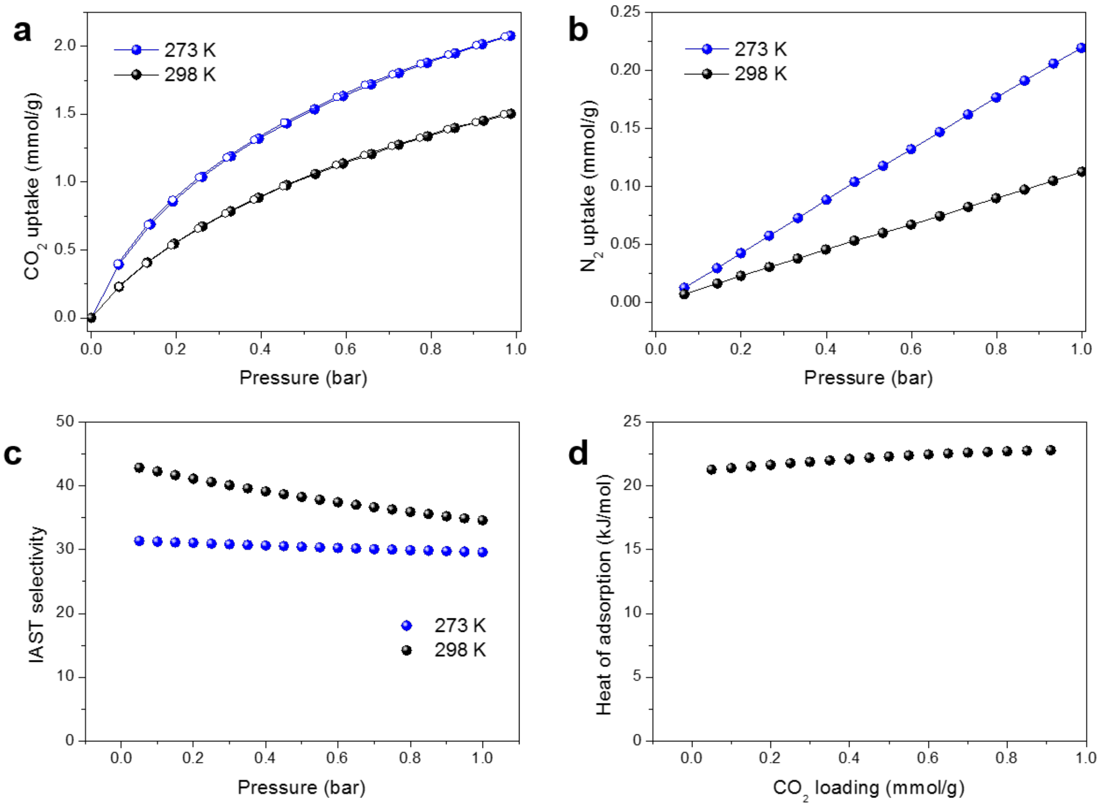

Supplementary figure 6 | Gas adsorption isotherms of COP-99; (**a**) CO_2 and (**b**) N_2 at 273 K and 298 K. (**c**) IAST CO_2/N_2 selectivity for CO_2:N_2 gas mixture (15:85). (**d**) Isosteric heat of CO_2 adsorption for COP-99.

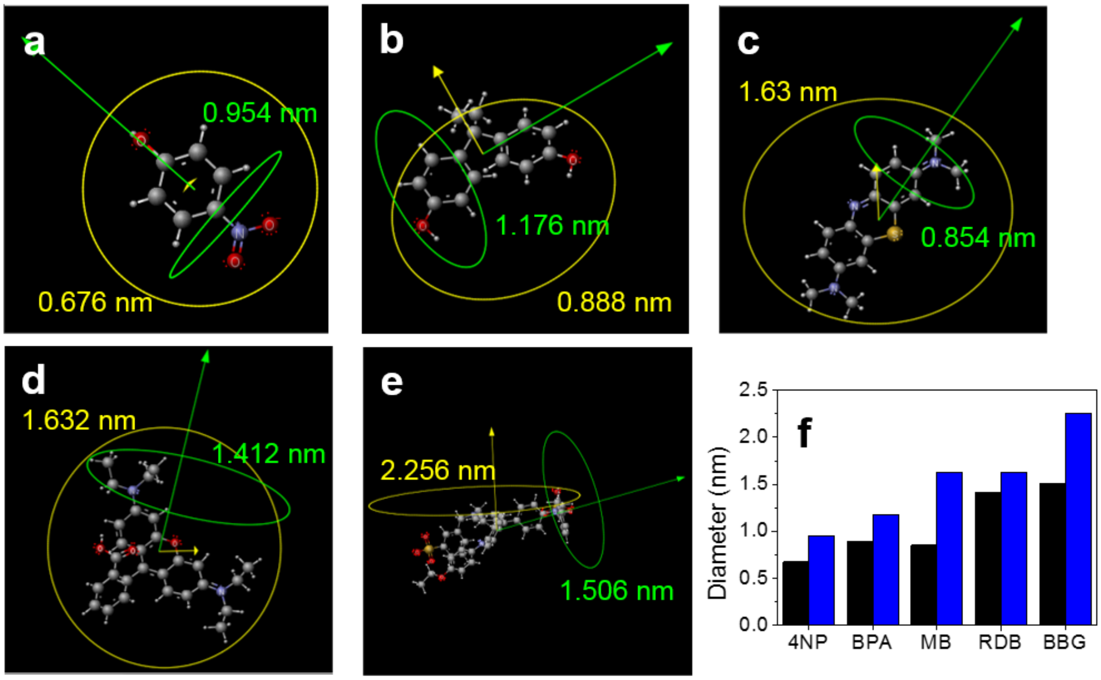

Supplementary figure 7 | Visualization of minimum (green) and maximum (yellow) projection diameter of dye molecules utilized for adsorption study; (**a**) 4-Nitrophenol (4NP), (**b**) Bisphenol A (BPA), (**c**) Methylene Blue (MB), (**d**) Rhodamine B (RDB), and (**e**) Brilliant Blue G (BBG). Arrows represent the orthogonal directions on the projection planes. (**f**) Calculated d_{min} (black) and d_{max} (blue) for dye molecules. The calculations were carried out using *MarvinSketch, ChemAxon* (version 15.6.8), for the molecular conformation with the lowest energy based on van der Waals diameter of the atoms. (Projection optimization enabled, optimization limit: very strict).

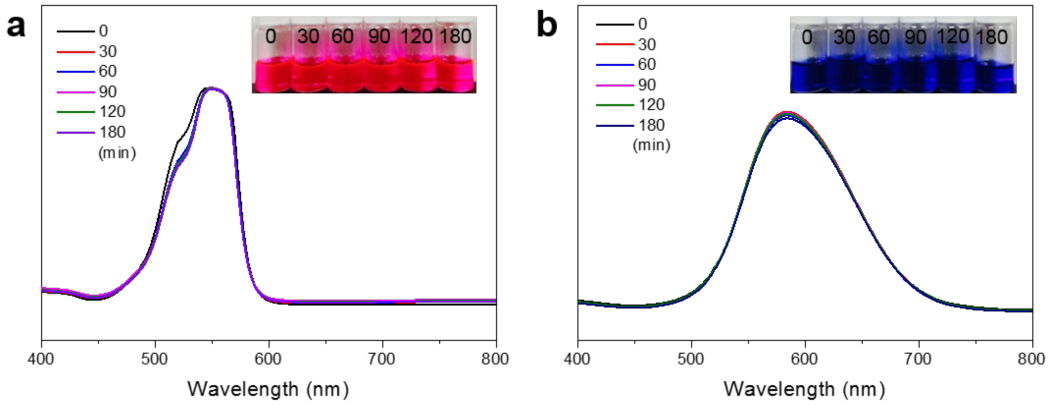

Supplementary figure 8 | UV-Vis absorption spectra of (**a**) aqueous RDB and (**b**) BBG treated with COP-99 at different intervals. The inset photograph shows the corresponding colour of the effluents after the treatment.

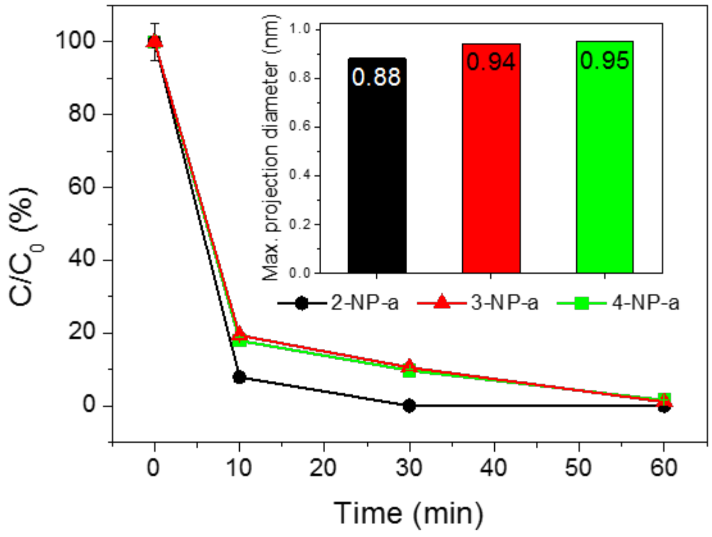

Supplementary figure 9 | Change in NP isomer concentrations over time after being treated with COP-99 in terms of absorbance relative to initial absorbance (C/C_0). Initial concentration (C_0) of all the dyes was adjusted to be 100 μM, and the adsorption was conducted in mild acid condition (pH ~3.8). Inset displays the maximum van der Waals diameter of NP isomers calculated by *MarvinSketch*.

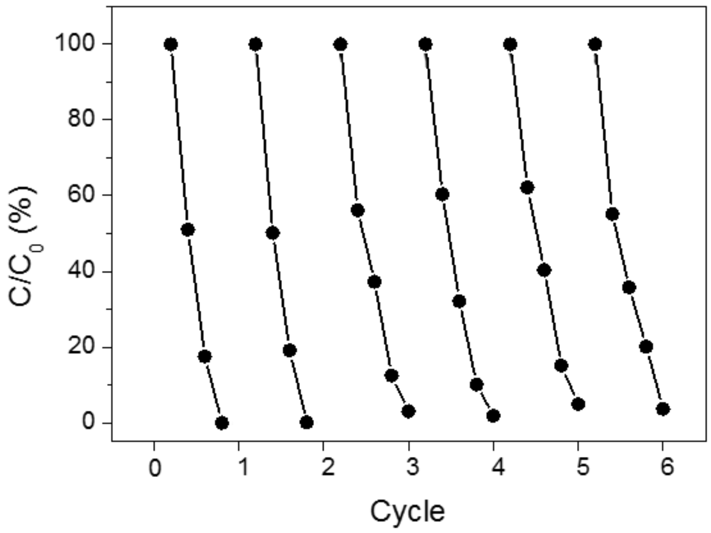

Supplementary figure 10 | Removal efficiency of 4-NP-a on COP-99 in six successive cycles of adsorption-desorption. Initial concentration of 4-NP-a was adjusted to be 50 μM and pH of all the samples was controlled to be 4.

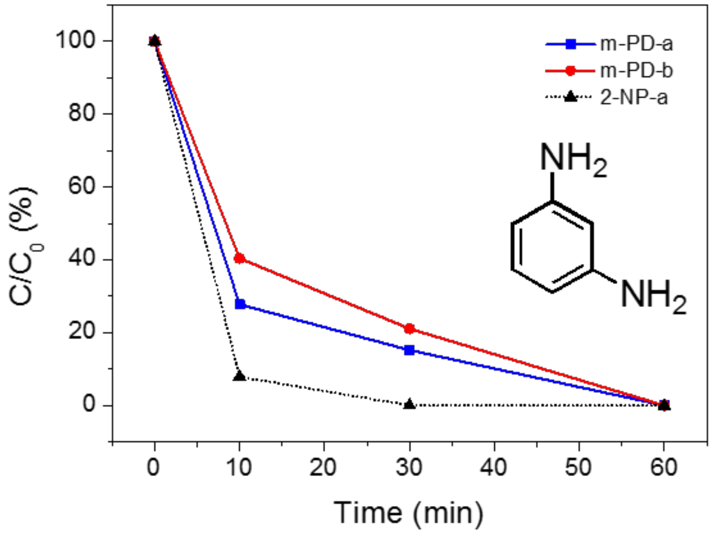

Supplementary figure 11 | Change in m-PD concentrations over time after being treated with COP-99 in terms of absorbance relative to initial absorbance (C/C_0). Initial concentration (C_0) of all the dyes was adjusted to be 100 μM. The m-PD was tested both in acidic (m-PD-a, pH = 4) and basic (m-PD-b, pH = 10) conditions. The concentration was analysed using a UV-vis spectrophotometer at a wavelength of maximum absorbance (270 nm for m-PD-a and 220 nm for m-PD-b) [10]. *Inset* shows molecular structure of m-PD.

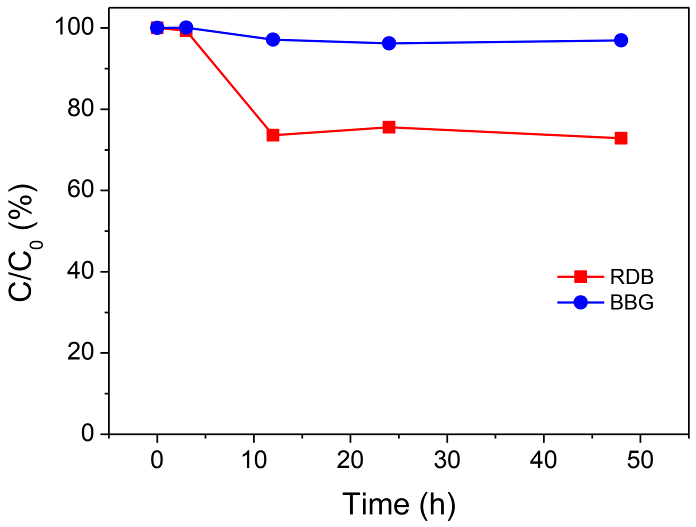

Supplementary figure 12 | Long-term adsorption test of RDB and BBG using COP-99. Initial concentration (C_0) of all the dyes was adjusted to be 50 μM.

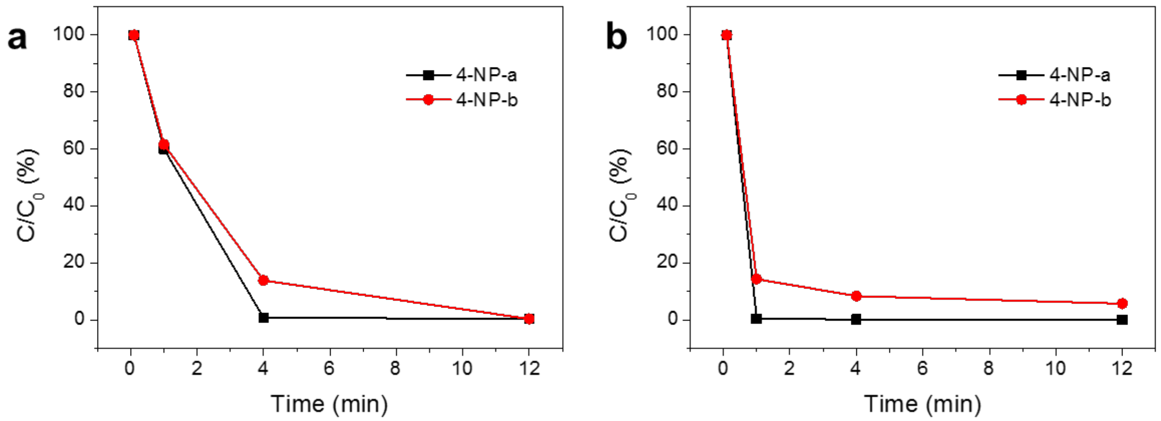

Supplementary figure 13 | Change in 4-NP concentrations over time after being treated with (**a**) CTF and (**b**) F-CTF in terms of absorbance relative to initial absorbance (C/C_0). Initial concentration (C_0) of the dyes was adjusted to be 50 μM. 4-Nitrophenol was tested both in acidic (4NP-a, pH = 4) and basic (4NP-b, pH = 9) conditions.

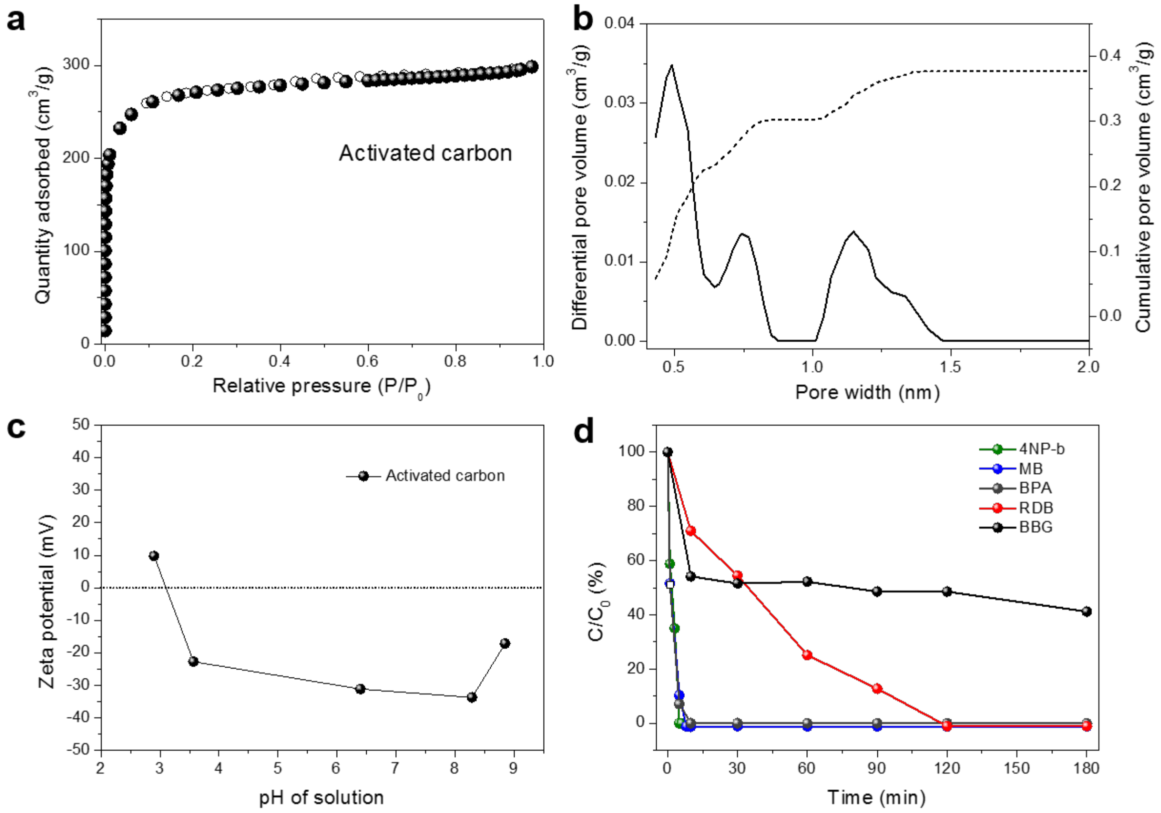

Supplementary figure 14 | Control experiments with activated carbon. (**a**) Argon adsorption-desorption isotherm of Activated Charcoal Norit® (ACN) measured at 87 K, and (**b**) corresponding NLDFT pore size distribution. (**c**) pH-dependent zeta potential of ACN. (**d**) Change in dye concentrations over time after being treated with ACN in terms of absorbance relative to initial absorbance (C/C_0). Initial concentration (C_0) of all the dyes was adjusted to be 50 μM.

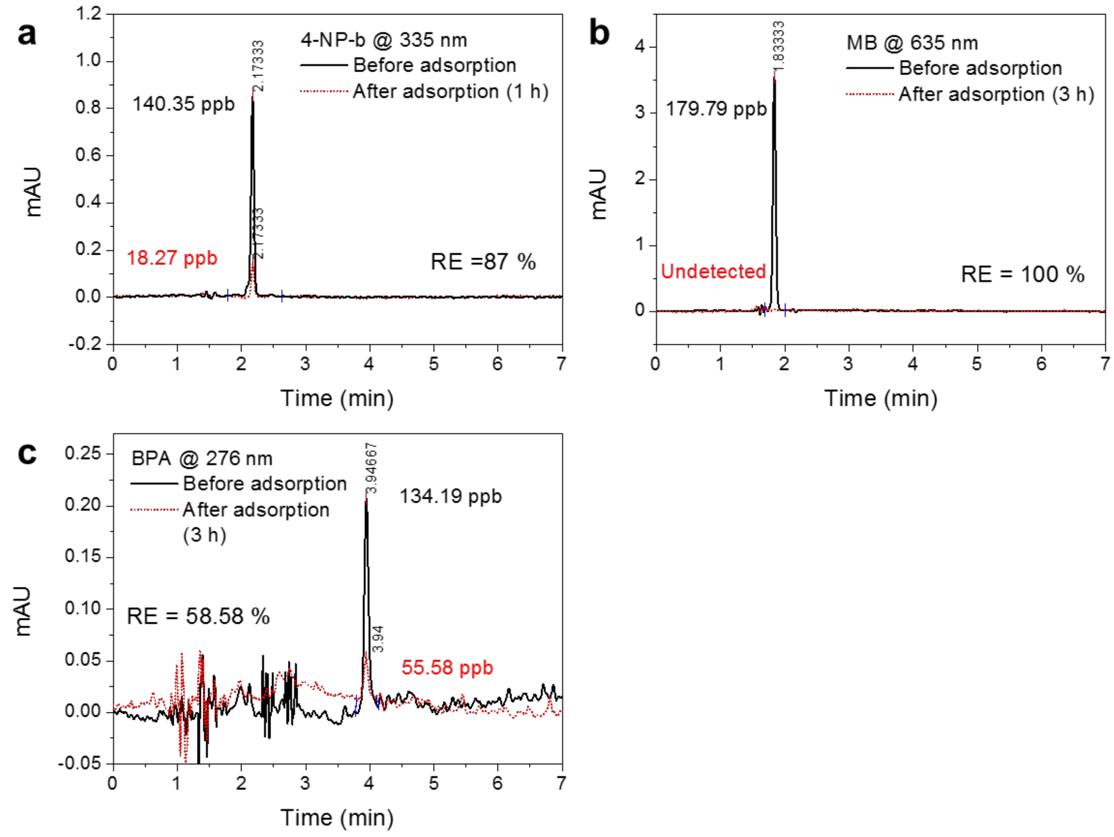

Supplementary figure 15 | LC spectra of (**a**) 4-NP-b, (**b**) MB and (**c**) BPA before and after the treatment with COP-99. RE stands for removal efficiency via the adsorption with COP-99.

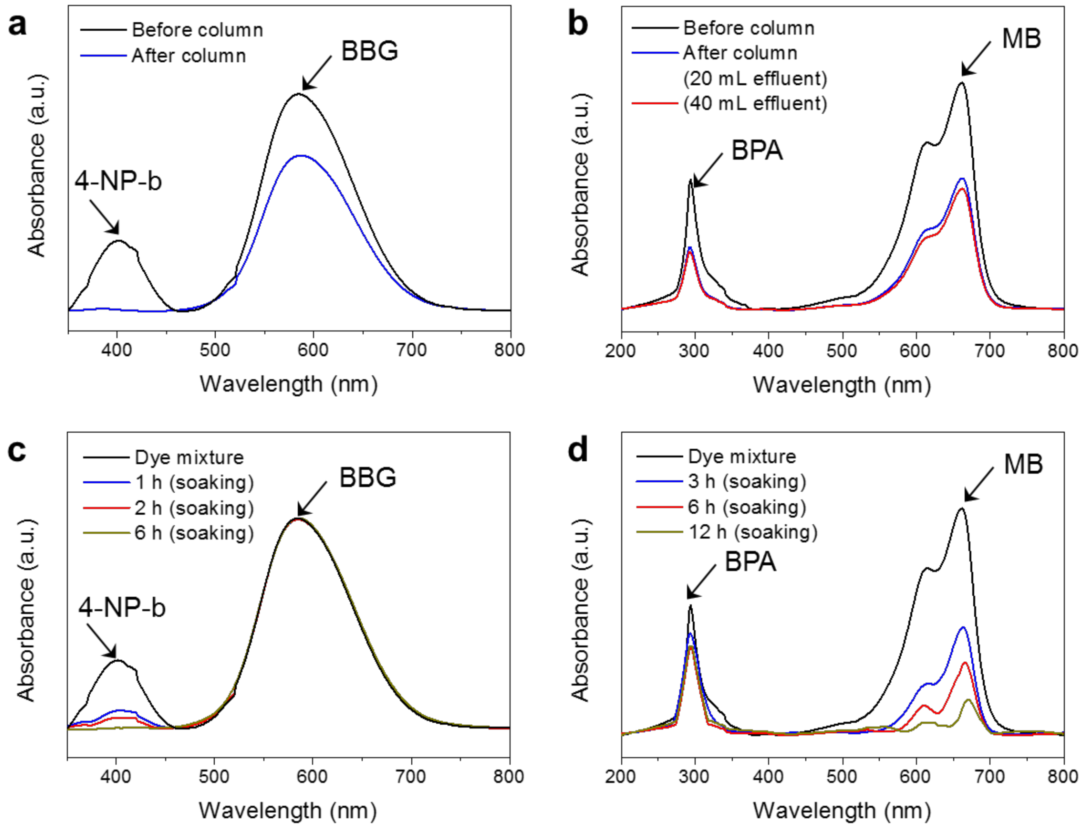

Supplementary figure 16 | UV-Vis spectra of mixed dye solutions of (**a,c**) 4-NP/BBG and (**b,d**) MB/BPA (**a, b**) before and after being treated via a packed column of COP-99 and (**c, d**) during soaking COP-99 in the solutions.

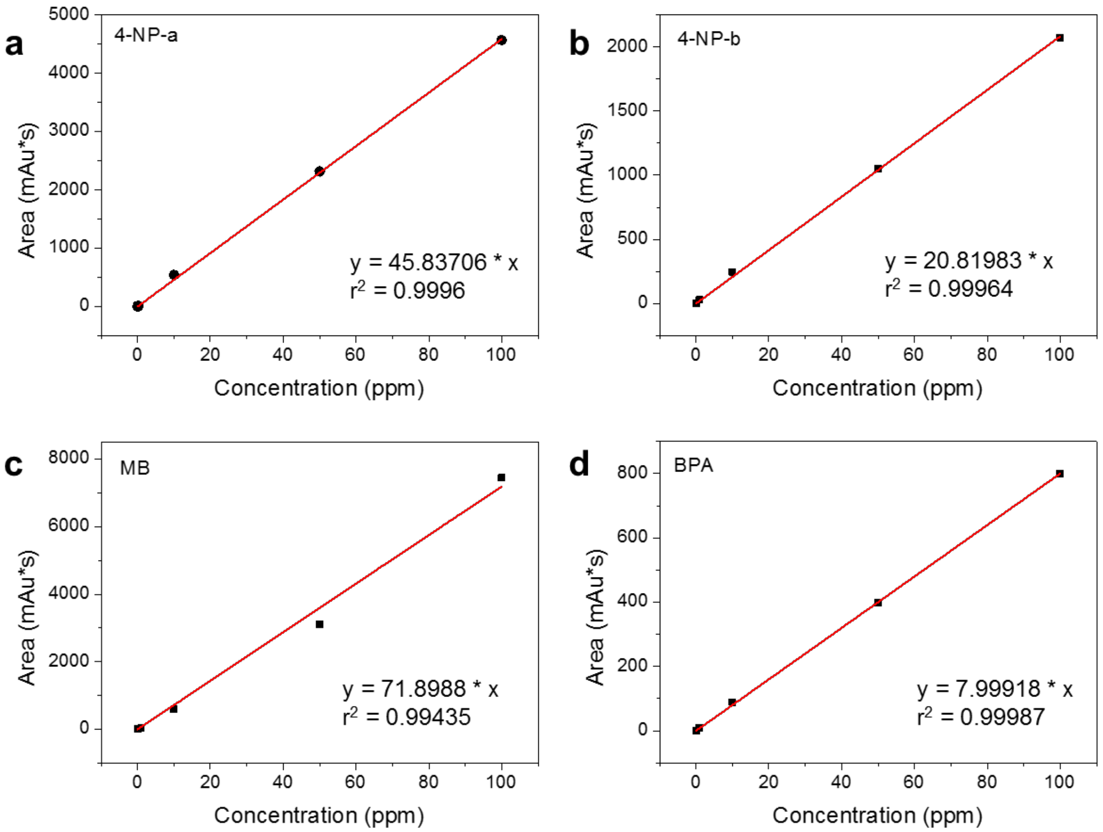

Supplementary figure 17 | HPLC calibration curves of the tested dye substrates from five concentrations of 0.05 ppm, 0.1 ppm, 10 ppm, 50 ppm, and 100 ppm. (**a**) 4-NP-a, (**b**) 4-NP-b, (**c**) MB, and (**d**) BPA.

Supplementary table 1 | Elemental analyses of all porous polymers. Fluorine contents were quantitatively measured by Combustion Ion Chromatography (CIC).

	Element	C	N	H	O	F
COP-99	Expected (%)	47.08	-	-	15.68	37.24
	Found (%)	50.05	0.92	0.87	26.73	21.51
F-CTF	Expected (%)	48.02	14.00	-	-	37.98
	Found (%)	60.16	21.26	2.82	-	15.82
CTF	Expected (%)	74.99	21.86	3.15	-	-
	Found (%)	67.49	17.93	2.38	6.7	-

Supplementary note 1. Long-term boiling test of COP-99 was carried out under hydrothermal condition. In order to prevent from the possible evaporation of D_2O under the elevated temperature, we carried out the test in closed glass ampoule. Typically, about 20 mg of COP-99 was placed in a Pyrex ampoule (5 mL capacity) and 1.5 mL of D_2O was transferred in the ampoule. The reaction mixture was frozen in liquid nitrogen, further evacuated and flame-sealed. After being warmed, the ampoule was treated at 110°C for 24h, and the reaction filtrate was analysed using NMR spectroscopy.

We observed that COP-99 did not exhibit any colour change and the D_2O filtrate also stayed transparent after the hydrothermal treatment (Supplementary Fig. 2d). The ^{1}H NMR spectrum of the D_2O filtrate only showed a D_2O residual peak, and the ^{13}C NMR spectrum also displayed no peaks, indicating the COP-99 is insoluble in water even at a boiling condition (Supplementary Fig. 2e and 2f, respectively). In the ^{19}F NMR spectrum (Supplementary Fig. 2g), however, showed a small peak at about -130.27 ppm, corresponding to aqueous F^- ions (e.g. Aqueous F- of KF = -125.3 ppm). This implies that few fluorines on COP-99 were detached from the polymer network at the elevated temperature, and we assumed that D_2O replaces fluorines to produce deuterium fluoride in high temperature via nucleophilic substitution. When COP-99 was treated under lower temperature, i.e., 50 °C for 60 h, there were no peaks in ^{1}H, ^{13}C, ^{19}F NMR spectrum in the D_2O filtrate (Supplementary Fig. 2h). We found that the fluorine content has been decreased 4.6 % after the boiling test at 110 °C (only 20 % of all fluorines) due to the fluorine exchange with D_2O, however, there was no noticeable change in fluorine content after the treatment at 50 °C. This indicates that the nucleophilic substitution of fluorines is plausible (albeit minor) under boiling condition, but the COP-99 was stable in water at least up to 50 °C with no change in the amount of fluorines and its framework is stable even at high temperature and pressures. Therefore, COP-99 should be safe to be utilized in water treatment application owing to its stability and insolubility in water.

Supplementary note 2. The CO_2 and N_2 uptakes of the COP-99 were carried out at 273 K and 298 K (Supplementary Fig. 6a and 6b). The CO_2 isotherms of COP-99 show physisorptive binding motion with good reversibility. The CO_2 adsorption capacity of COP-99 at 1 bar is 2.14 mmol g^{-1} at 273 K and 1.55 mmol g^{-1} at 298 K, showing noticeable capacity retention with rising temperature. The CO_2 uptake of COP-99 under the high temperature is highly comparable to fluorinated porous polymers with much higher BET surface area, such as FPOP (2.2 mmol g^{-1} at 273 K, surface area = 1170 m^2 g^{-1})[5] and FMOP (1.68 mmol g^{-1} at 298 K, surface area = 1018 m^2 g^{-1})[6], which may be originated from the high fluorine content and narrower pore of COP-99 facilitating CO_2 adsorption[3,7]. The preferential CO_2 binding capability of COP-99 was evaluated by using an ideal adsorbed solution theory (IAST) for binary mixture composition of CO_2:N_2 (15:85)[8]. The IAST CO_2 and N_2 selectivity was high as 29.6 at 273 K and 34.6 at 298 K, showing a slight enhancement in increasing temperature (Supplementary Fig. 6c). Such behaviour is due to CO_2 binding affinity toward the fluorinated COP-99 not weakening as much as the N_2 binding[9]. The isosteric heat (Q_{st}) of COP-99 for CO_2 adsorption was 21.3 kJ mol^{-1} at zero coverage (Supplementary Fig. 6d), indicating that COP-99 can be easily regenerated via pressure/vacuum swing adsorption technique.

Supplementary note 3. Along with 4-NP/BBG mixture shown in Fig. 7b, mixture of MB and BPA having similar molecular size was also tested for column separation and batch adsorption. When MB/BPA mixture was filtered through the COP-99 column, both molecules exhibited concentration decrease in a similar level, showing the removal efficiency of 47 % and 55.4 % for MB and BPA, respectively (Supplementary Fig. 16b). We believe this is originated from the column packing condition as we have seen the separation test result from 4-NP/BBG mixture. The size-dependent separation was dominant than the charge-based separation when the mixed molecules pass through a densely packed column, and MB and BPA having almost similar molecular size were filtered out at the same time. While there was no selective adsorption in the column separation, COP-99 showed charge-selective uptake of MB out of the MB/BPA dye mixture via batch adsorption. After immersing 8 mg of COP-99 in the 8 mL of MB/BPA mixed solution for 12 h, MB was removed up to 87 % and BPA was adsorbed 22 %, indicating the fluorine-charge interaction facilitates selective adsorption of MB from the mixed solution (Supplementary Fig. 16d).

Supplementary references

1. Budd PM, *et al.* Solution-processed, organophilic membrane derived from a polymer of intrinsic microporosity. *Adv. Mater.* **16**, 456-459 (2004).

2. Kuhn P, Antonietti M, Thomas A. Porous, covalent triazine-based frameworks prepared by ionothermal synthesis. *Angew. Chem. Int. Ed.* **47**, 3450-3453 (2008).

3. Zhao YF, Yao KX, Teng BY, Zhang T, Han Y. A perfluorinated covalent triazine-based framework for highly selective and water-tolerant CO_2 capture. *Energy Environ. Sci.* **6**, 3684-3692 (2013).

4. Clark T, Hennemann M, Murray J, Politzer P. Halogen bonding: the σ-hole. *J. Mol. Model.* **13**, 291-296 (2007).

5. Liu DP, Chen Q, Zhao YC, Zhang LM, Qi AD, Han BH. Fluorinated Porous Organic Polymers via Direct C-H Arylation Polycondensation. *ACS Macro Lett.* **2**, 522-526 (2013).

6. Yang ZZ, *et al.* Fluorinated microporous organic polymers: design and applications in CO_2 adsorption and conversion. *Chem. Commun.* **50**, 13910-13913 (2014).

7. Li GY, Zhang B, Yan J, Wang ZG. The directing effect of linking units on building microporous architecture in tetraphenyladmantane-based poly(Schiff base) networks. *Chem. Commun.* **50**, 1897-1899 (2014).

8. Myers AL, Prausnitz JM. Thermodynamics of mixed-gas adsorption. *AIChE J.* **11**, 121-127 (1965).

9. Patel HA, Ko D, Yavuz CT. Nanoporous Benzoxazole Networks by Silylated Monomers, Their Exceptional Thermal Stability, and Carbon Dioxide Capture Capacity. *Chem. Mater.* **26**, 6729-6733 (2014).

10. Baranowska I, Pieszko C, Raróg D, Pielesz A. Analysis of some aromatic amines by means of derivative spectrophotometry. *J. Environ. Sci. Heal. A* **37**, 1841-1848 (2002).
